# HATRIC: a study of *Pelargonium sidoides* root extract EPs®7630 (Kaloba®) for the treatment of acute cough due to lower respiratory tract infection in adults—study protocol for a double blind, placebo-controlled randomised feasibility trial

**DOI:** 10.1186/s40814-019-0478-6

**Published:** 2019-07-31

**Authors:** Amy Whitehead, Catherine Simpson, Merlin Willcox, Frances Webley, Alastair D. Hay, Chris Butler, Lily Yao, Emma Wrixon, Margaret Bell, Jennifer Bostock, Paul Little, Gareth Griffiths, Michael Moore

**Affiliations:** 1 Southampton Clinical Trials Unit, Faculty of Medicine, University of Southampton MP131, Southampton General Hospital, Tremona Road, Southampton, SO16 6YD UK; 2 Primary Care and Population Science, Faculty of Medicine, University of Southampton, Aldermoor Health Centre, Southampton, SO16 5ST UK; 3 Centre for Academic Primary Care, Population Health Sciences, Bristol Medical School, University of Bristol, Bristol, BS8 2PS UK; 4 Nuffield Department of Primary Health Care Sciences, University of Oxford, Oxford, OX2 6GG UK; 5 Department of Health Sciences, University of Leicester, Leicester, LE1 7RH UK

**Keywords:** Feasibility, Herbal, Double-blind randomised, Retention, Variance, Stratification, Lower respiratory tract, Acute bronchitis, Cluster randomised

## Abstract

**Background:**

Acute lower respiratory tract infection is a common acute infection managed in primary care. The current dominant management strategy in the UK is antibiotics, despite widespread publicity regarding antimicrobial resistance and evidence that the small benefits of antibiotics do not outweigh the harms. There is a need to address the rising problem of antibiotic resistance by providing credible alternative strategies, which reduce symptom burden. There is sufficient evidence to recommend the use of *Pelargonium sidoides* root extract in order to warrant undertaking an independent clinical trial.

We propose a feasibility study to demonstrate our ability to recruit and retain patients and conduct a placebo-controlled trial of *Pelargonium sidoides* extract EPs®7630 in lower respiratory tract infection where pneumonia is not suspected. Both the tablet and liquid formulations will be included.

**Methods:**

The HATRIC trial is a double-blind randomised placebo-controlled feasibility study aiming to determine the potential to conduct a fully powered trial of *Pelargonium sidoides* root extract as an alternative to the inappropriate use of antibiotics for acute bronchitis in UK primary care.

Primary care sites will be equally randomised to one of two formulation groups (tablet or liquid preparation). Additionally, within each site, patients will be evenly randomised to active or placebo treatment. Antibiotic consumption will be monitored during the trial, but the use of a delayed prescription strategy is encouraged. The target sample size for this study is 160 patients overall or 40 per arm, recruited from approximately 20 primary care sites. The analysis will be descriptive focusing on estimation with no formal comparison of groups taking place.

**Discussion:**

If this trial demonstrates the feasibility of recruitment and delivery, we will seek funding for a fully powered placebo-controlled trial of *Pelargonium sidoides* root extract for the treatment of lower respiratory tract infections in primary care.

**Trial registration:**

HATRIC was registered on the ISRCTN registry (ISRCTN17672884) on 16 August 2018.

## Background

Acute non-pneumonic lower respiratory tract infection (LRTI)/acute bronchitis is a common acute infection managed in primary care. A systematic review of the use of antibiotics for acute bronchitis suggested modest benefits; however, the authors suggested that any modest benefits that accrued were matched by harm from side effects from antibiotics [1]. In the largest of the contributing studies [2], no subgroups were identified who derived clinically relevant benefit from antibiotics [3]. Avoidance of unnecessary antibiotic prescriptions is one of the key components of the UK’s national action plan on tackling antimicrobial resistance [4, 5].

Antibiotics are still widely and inappropriately prescribed for acute bronchitis in the UK with no sign of substantial reduction despite widespread publicity regarding antimicrobial resistance [6–8]. Various strategies have been proposed in national guidelines including delayed prescribing [9] and wider use of the C-reactive protein (CRP) test [10] but have yet to have substantial impact. The symptoms of acute bronchitis are troublesome, and cough is known to persist for around 3 weeks on average [2, 11]. One alternative approach is to provide symptom relief, which in conjunction with a delayed prescription if effective may substantially reduce antibiotic uptake. The impact of effective alternative treatments for respiratory infections is likely to be in minimising unnecessarily severe or prolonged symptoms and avoiding side effects from unnecessary antibiotics; minimising the long-term public health risks of inappropriate antibiotics and providing a model for different management strategies for other respiratory tract infections (RTIs).

Some potential candidates for symptom relief such as steam inhalation have not been shown to be helpful [12] whilst ibuprofen had no significant benefit [13] and may cause harm [12]. Other potential symptomatic treatments in adults (the expectorant guaifenesin, mucolytics, and antihistamine-decongestant combinations) have not been shown to have consistent benefit in a recently updated Cochrane systematic review [14].

In a systematic review of herbal treatments for acute upper respiratory infection and cough, there was supporting evidence for *Andrographis paniculata* and *Pelargonium sidoides* [15]. A Cochrane review updated in 2013 provided supporting evidence for *Pelargonium sidoides* root extract EPs®7630 in acute respiratory infections in both adults and children [16], and although this is suggested for self-care in the latest draft NICE guidance [17], the quality of the evidence was regarded as very low, suggesting the need for more robust research.

*Pelargonium sidoides* root extract EPs®7630 is widely available over the counter in several countries in Europe, Asia, and Central and South America as well as in Australia. A variety of potential mechanisms have been identified for EPs®7630 which provide a rational basis for its use, including moderate antiviral and antibacterial effects and the possession of immune-modulatory capabilities that are mainly mediated by the release of tumour necrosis factor α and nitric oxides, the stimulation of interferon-β, and an increase in natural killer cell activity [18–21]. Moreover, EPs®7630 has been shown to activate human monocytes, induce mitogen-activated protein kinase-dependent pro-inflammatory cytokines in these cells, and specifically modulate their production capacity of mediators that are known to lead to an increase of acute phase protein production in the liver, neutrophil generation in the bone marrow, and the generation of adaptive T-helper 17 and T-helper 22 cells. An improved phagocytosis, oxidative burst, and intracellular killing by human peripheral blood phagocytes as well as an inhibition of group-A streptococci and host epithelia [19, 21] and an increase of stress resistance [22] was also shown. *Pelargonium sidoides* root extract EPs®7630 interferes with replication of seasonal influenza A virus strains (H1N1, H3N2), human coronavirus, RSV, parainfluenza virus, and coxsackie virus, whilst no direct virucidal effect was observed [23]. Exertion of anti-influenza virus activity was also confirmed in an animal model [24]. The data available therefore constitute sufficient evidence to warrant undertaking a high-quality independent clinical trial of this herbal medicine.

We propose a feasibility study to demonstrate our ability to recruit and retain patients with acute bronchitis in a placebo-controlled trial of *Pelargonium sidoides* extract EPs®7630. Furthermore, we propose to include both liquid and tablet formulations and to use a mixed methods approach to critically examine recruitment and retention issues prior to proceeding with a fully powered trial.

## Methods/design

The HATRIC (Herbal Alternative Treatment (Pelargonium) for Lower Respiratory Tract Infections with Cough in adults) trial (ISRCTN17672884; sponsor reference number: 24375) is a double-blind, randomised placebo-controlled feasibility study, with general practitioner (GP) practices cluster-randomised to give liquid or tablet preparation, and within each practice, eligible patients randomised to *Pelargonium sidoides* root extract EPs®7630 or placebo.

A qualitative study, HATRIC-Q (IRAS project ID: 233467; sponsor reference number: 29921), will also be undertaken with patients, including both those who agreed and those who declined to take part in HATRIC, and clinicians. We will explore their experiences of taking part in the trial and obtain their feedback on trial procedures, as well as their views on the implementation of a delayed antibiotic approach and the use of herbal medicine for the treatment of lower respiratory tract infection. This qualitative sub-study is described fully in a separate protocol.

### Objectives

The aim of this study is to determine the feasibility of conducting a fully powered trial of *Pelargonium sidoides* root extract as an alternative to inappropriate use of antibiotics for lower respiratory tract infections in UK primary care. Specific feasibility objectives are detailed below (see Table 1).

**Table 1:** Feasibility objectives and endpoints for the HATRIC trial

Feasibility objective	Endpoint used to evaluate
Eligibility: number of patients included and number excluded (+ reasons) from the trial	On site screening logs
Recruitment: ability to recruit patients into the intervention from those attending primary care	On site enrolment records—monthly rate/site adjusted for site list size
Randomisation: willingness to be randomised	Proportion of eligible patients recruited
Retention: across the duration of the intervention and return of a fully completed diary	Quantitative data from enrolment Withdrawal rate from study Completion of outcome measures
Intervention compliance	Diary data and returned medication
Patient preference for liquid/tablet formulation	Diary data Returned medication Recruitment data
Establish the frequency of collecting EQ5D questionnaire and identifying the key resources associated with the intervention and potential influence on service usage Establish methods of data collection for the health economics analysis	Development of health economic protocol and collection of outcome data EQ-5D-5L questionnaire and case note review
Acceptability of the patient diaries, patients’ willingness to complete them, and the importance of telephone/text contact	Quantitative data collection—percentage of patients returning completed diaries
Success of delayed antibiotic strategy	Diary data on day antibiotics commenced
Need for stratification by antibiotic strategy in main study	Proportion allocated to immediate and delayed antibiotic strategy
To inform sample size for future trials	Rate of outcome measures in the control group
Quality of life measurements and resource use	Identify the key resource items to be collected and how often to collect EQ-5D-5L in the full trial

### Study participants and enrolment

The inclusion and exclusion criteria for the HATRIC trial are listed in Table 2. Up to twenty health centres in the Wessex region of the UK will identify eligible patients and will invite them to participate in the trial.

**Table 2:** Eligibility criteria for the HATRIC trial

Inclusion criteria	
1. Adults 18 years and over	
2. Presenting with an acute cough (≤ 21 days’ duration) as their main symptom	
3. Presenting with symptoms localising to the lower tract (e.g. sputum, chest pain, dyspnoea, wheeze), for which an infective diagnosis is judged very likely	
4. Willing and able to give written informed consent	
Exclusion criteria	
1. Suspected pneumonia (i.e. complicated lower respiratory tract infection) on the basis of focal chest signs (focal crepitations, bronchial breathing) and systemic features (severe breathlessness, high fever, vomiting, severe diarrhoea)	
2. Signs of severity which may warrant hospital admission (e.g. SpO2 < 91% [30, 31], Systolic BP < 90 mmHg, Heart rate > 130)	
3. Exacerbation of COPD	
4. Serious chronic disorders where immediate antibiotics are needed (e.g. cystic fibrosis, valvular heart disease)	
5. Unable to give informed consent or complete trial paperwork (including the patient diary)	
6. Difficulty reading and understanding English and therefore unable to give informed consent or complete the trial paperwork (including the patient diary)	
7. Known or suspected pregnancy	
8. Women at risk of pregnancy (i.e. not on effective contraception—combined oral contraceptive pill, an intrauterine hormonal device, subcutaneous hormonal implant, or hormonal contraceptive injections)	
9. Currently breast-feeding	
10. Known immunodeficiency state or chemotherapy	
11. Currently taking oral steroids	
12. Using a *Pelargonium sidoides*/Kaloba® preparation and unwilling or unable to discontinue for the study period	
13. Hypersensitivity to *Pelargonium sidoides* preparations or to the Kaloba brand	
14. Increased tendency to bleeding or is taking coagulation-inhibiting drugs (e.g. warfarin)	
15. Severe hepatic and renal diseases (Chronic Kidney Disease Stage 4, GFR < 30), as no adequate data are available in these areas	
16. Rare hereditary problems of galactose intolerance, the Lapp lactase deficiency, or glucose-galactose malabsorption (tablet formulation only)	
17. Previously entered the HATRIC trial	
18. Recruited to another interventional trial in the previous 6 weeks	

They will be given a full explanation of the trial, an information leaflet, and allowed sufficient time to decide whether to participate and ask any questions they may have. Only then will written informed patient consent be obtained by the recruiting GP or nurse or by appropriately trained healthcare assistants or clinical trials assistants. The right of the patient to refuse to participate without giving reasons will be respected. After the patient has entered the trial, the clinician remains free to give alternative treatment to that specified in the protocol at any stage if he/she feels it is in the patient’s best interest, but the reasons for doing so will be recorded. In these cases, the patients will remain within the trial for the purposes of follow-up and data analysis. All patients will be free to withdraw at any time from the protocol treatment without giving reasons and without prejudicing further treatment.

All eligible patients including those who decline to be involved in the trial will be invited to consider being interviewed for the HATRIC-Q study. Those entering the trial will be asked to provide consent for their contact details to be shared with the qualitative researcher. Those who decline participation in the HATRIC trial will be given an invitation letter for HATRIC-Q, and if interested, they will contact the qualitative researcher directly.

Women of childbearing potential will be required to use one of the most effective contraceptive methods (combined oral contraceptive pill, hormonal intrauterine device, hormonal contraceptive injection, or subcutaneous hormonal implant) to enter the trial. There are no safety data available for pregnant women, and therefore, the manufacturing authorisation in Germany for the Kaloba® preparation specifies that it must not be taken by women who are pregnant or breastfeeding.

Screen failures will be documented in the screening log maintained at each participating site, together with reasons for exclusion.

Patients who are not eligible or who declined to take part in the HATRIC trial will be given, by the GP, nurse, or research assistant, a letter with a reply slip inviting them to participate in HATRIC-Q.

### Randomisation

Participating primary care sites will be randomised to either tablet or liquid preparation in a 1:1 allocation ratio. GPs and patients will know whether they are receiving tablets or liquid, as this cannot be blinded.

In each site, patients who meet the eligibility criteria for the study and for whom written informed consent can be obtained will be individually randomised to either the active or the placebo treatment of either the tablet or the liquid form depending on the site allocation. This individual randomisation will be block randomised (with varying block size) in a 1:1 allocation ratio of placebo to active treatment, and no stratification factors will be used.

The treatment packs will be sent to sites in sets of four, and each patient will be allocated the next available sequentially numbered patient pack at their site. The doctor or nurse allocating the patient pack and the patient will not know to which arm they have been randomised (placebo or EPs®7630). The patient pack will contain either *Pelargonium sidoides* root extract EPs®7630 in liquid or tablet form OR matching placebo.

### Trial procedures

#### Baseline

At the initial visit the patient’s relevant past medical history, baseline symptoms, and vital signs will be recorded.

Patient’s baseline symptoms—The severity of the listed symptoms will be scored according to the following system: 0 = no problem, 1 = very little problem, 2 = slight problem, 3 = moderate problem, 4 = bad problem, 5 = very bad problem, and 6 = as bad as it could be. The symptoms captured will be cough, phlegm, shortness of breath, wheeze, blocked or runny nose, chest pain, muscle aches, headaches, disturbed sleep, general feeling of being unwell, fever, and interference with normal activities.

Patient’s vital signs—The following patient vital signs will be recorded: blood pressure; heart rate; temperature; oxygen saturation levels (Sats); C-reactive protein (CRP), if measured; and presence of wheeze or crackles in the chest.

As part of the baseline data capture, patients will also fill in the first section of the patient diary which contains questions about sociodemographic factors, such as their occupation, employment, and ethnicity; smoking; relevant medical history; present illness; and expectations about antibiotic treatment.

Patients will receive a £5 shopping voucher on entering the trial and a second £5 voucher when they have returned a fully completed diary or have completed a symptom diary by recall over the telephone as a thank you for participating. Contact details will be collected from all patients to enable follow-up phone calls to be made and shopping vouchers to be sent by the research team at the Southampton Clinical Trials Unit (SCTU).

#### Follow-up

##### Patient symptom diary

Patients will complete daily diary data for up to 28 days after presentation. They will be asked to stop completing the diary 2 days after complete resolution of symptoms. The collection of diary scores will not be limited to the time during which study medication is being used, to allow the capture of total symptom duration. The diary has previously been validated and is sensitive to change and internally reliable [25, 26].

Patients will also keep a record of the number of times trial medication has been taken every day and, if applicable, when antibiotics or other treatments for their chest infection were taken. Patients will also be asked questions about resource use for their acute bronchitis, including consultations with healthcare professionals in secondary care, medications purchased, and absences from work.

All patients will complete the EQ-5D-5L questionnaire at baseline (day 1) and day 7, and half of the patients will also be randomly selected to complete the questionnaire at additional time points on day 2 and day 4. This will allow us to assess the acceptability of the frequency in collecting quality of life data.

After recruitment, patients will be contacted after 1–2 days to check for any problems with diary completion. Patients will be contacted again after 14 and 28 days to prompt diary completion and return. In the event of diaries not being returned, a brief telephone interview to collect the key data will be undertaken after 35 days. Patients will also be contacted in the event that a diary is returned incomplete, to obtain missing key data.

Contact with patients at each time point will be made initially by text or email, if the patient has provided a mobile telephone number or an email address, to explain when we will be calling and then by telephone.

Completed diaries will be returned to the Southampton Clinical Trials Unit in a freepost envelope. To improve the return rate of completed diaries, patients who return a fully completed diary or complete a diary by recall will receive a £5 shopping voucher to thank them.

##### Notes review

A review of medical records will be undertaken to document return visits to GP practices/walk-in centres and out-of-hours clinics with a respiratory infection in the 28 days following recruitment. In addition, resource use information will be extracted for each patient including medication, primary care visits, outpatient visits, and hospital admissions.

All trial procedures are depicted in the SPIRIT figure [32] shown in Table 3.

**Table 3:** SPIRIT figure for HATRIC trial

Observation/procedure	Person undertaking the specified event	Timings of visit/contact				
		Screening/registration D1	D2-3	D14	D28	D35
Informed consent	GP/nurse^1^/HCA^1^/CTA^1^	X				
Inclusion/exclusion criteria	GP/nurse prescriber^1^	X				
Baseline symptoms^2^	GP/nurse	X				
Relevant medical history	GP/nurse	X				
Vital signs^3^	GP/nurse	X				
Issue trial medication	GP/nurse	X				
Issue patient diary	GP/nurse	X				
Completion of patient diary (days 1–28)	Patient	X	X	X	X	
Phone call to patient (diary assessment)	SCTU		X	X	X	Only if diary not returned or incomplete
Completion of diary by recall	Patient/SCTU					X
Adverse event notification^4^	Patient/SCTU		X	X	X	
Adverse event (AE) assessing	GP		X	X	X	
AE recording/reporting^4^	GP/nurse		X	X	X	
Concomitant medication (only to be recorded in the event of an SAE and specified AEs)	GP/nurse		X	X	X	
SAE assessing	GP		X	X	X	
SAE reporting	GP/nurse		X	X	X	
Medical note review	GP/nurse				X	

^1^In line with local GP surgery procedures with demonstrable and appropriate level of training. Specific duties delegated by the PI

^2^The severity of cough, phlegm, shortness of breath, wheeze, blocked or runny nose, chest pain, muscle aches, headaches, disturbed sleep, general feeling of being unwell, fever, and interference with normal activities

^3^Blood pressure; heart rate; temperature; oxygen saturation levels (Sats); C-Reactive protein (CRP), if measured; and the presence of wheeze or crackles in the chest

^4^Patients will be asked to notify their GP/nurse of specified adverse events. In addition, specified adverse events that are discovered by SCTU staff during patient contact phone calls will be notified immediately to GP/nurse. Reporting and recording of all AEs is carried out by GP/nurse. NB: the Patient is free to withdraw consent at any time without providing a reason. When withdrawn, the patient will continue to receive standard clinical care. Follow-up data will continue to be collected (unless the patient has specifically stated that they do not want this to happen)

#### Trial discontinuation

In consenting to the study, patients will have consented to the study intervention, follow-up, and data collection. Patients may be discontinued from the study procedures at any time.

Patients may be discontinued from the study in the event of:

- Clinical decision, as judged by the principal investigator
- The development of toxicity, regardless of causality, which, in the investigator’s opinion, precludes further treatment under this protocol
- The patient withdraws consent
- The recruiting physician’s judgement due to medical reason, e.g. concurrent illness, pregnancy.
- Non-compliance with protocol

Full details of the reason for trial discontinuation will be recorded in the end of study electronic case report form (eCRF) and medical record. Recruiting physician’s judgement refers to the discontinuation of patients due to a clinical judgement made post-randomisation but whilst carrying out the recruitment and baseline trial processes for a patient, for example, if a patient is found to be ineligible whilst having their full history taken and examination carried out. At any other time within the trial, a patient discontinued due to clinical judgement should be listed as a discontinuation due to a clinical decision, for example, if a patient develops symptoms that could be a side effect of the trial medication.

#### Withdrawal

The patient will be free to withdraw consent from the study at any time without providing a reason. Investigators will explain to patients the value of remaining in trial follow-up and allowing this data to be used for trial purposes so that where possible, patients who have withdrawn from trial treatment will remain in follow-up as per the trial schedule. If patients additionally withdraw consent for this, they will revert to standard clinical care as deemed by the responsible clinician. It would remain useful for the trial team to continue to collect standard follow-up data, and unless the patient explicitly states otherwise, follow-up data will continue to be collected. Details of withdrawal (date, reason if known) will be recorded in the end of study eCRF and medical record.

#### End of trial

The end of the trial will be when the last patient has had their last data that answers the research question collected and verified.

#### Unblinding procedures

Any emergency clinical decisions required will not be affected or altered by knowledge of the treatment group allocated to the patient. If unblinding is required, this can be done by the trial statisticians at the SCTU.

### Treatments

Primary care sites will be randomised to one of two groups (tablet or liquid preparation), and within each site, patients will be randomised to active or placebo IMP.

This will result in four treatment groups:

1. Liquid *Pelargonium sidoides* root extract EPs®7630—30 drops 3× daily, to be taken 30 min before meals
2. Liquid placebo—30 drops 3× daily, to be taken 30 min before meals
3. Tablet *Pelargonium sidoides* root extract EPs®7630—one 20 mg tablet 3× daily, to be taken 30 min before meals
4. Tablet placebo—one 20 mg tablet 3× daily, to be taken 30 min before meals

The tablets, liquid, and matching placebos will be provided and packaged ready for distribution to sites by Dr. Willmar Schwabe GmbH & Co. KG, Karlsruhe (Germany).

Patients will be asked to take the IMP daily until 2–3 days after symptoms have resolved, but treatment duration should not exceed 2 weeks. If a dose is missed, the patient should not take twice the dose but continue to take their usual dose at the usual time.

The use of the delayed prescription strategy for antibiotics will be encouraged (because it reduces use of antibiotics whilst maintaining patient satisfaction [27]), but clinicians will be able to offer one of three following antibiotic strategies in addition to the randomised intervention:

1. Immediate antibiotics
2. Delayed antibiotics
3. No antibiotics

No class of antibiotic has been shown to have superior performance to another in the treatment of LRTI/acute bronchitis, and hence, the prescribing physician will prescribe delayed antibiotics according to local guidelines. Patients will be asked to wait 7–10 days before collecting the delayed prescription unless their symptoms show substantial deterioration.

### Accountability

HATRIC trial medication will be stored centrally and distributed to GP sites by the SCTU. Drug accountability logs will be maintained by the SCTU and at individual sites. Additionally, patients will be asked to record trial medication usage in their patient diary and to return all unused IMP and packaging to the research team.

### Data collection

The majority of patient data will be entered by research staff at site via a remote data collection tool (Medidata Rave). Diary data will be entered into the trial database by the research team. All data will be retained in accordance with the General Data Protection Regulations. The PI is responsible for ensuring the accuracy, completeness, and timeliness of the data entered.

Data will be checked for missing or unusual values (range checks) and checked for consistency within patients over time by SCTU staff. Any discrepant data collected at site will be returned to the site in the form of electronic data queries. Queries regarding the diary data will be discussed with patients as outlined above in the patient follow-up section. Full details on data management procedures are available in the HATRIC Data Management Plan, available on request.

All other processes relating to the storing of trial data, trial monitoring and audit, and archiving will follow standard GCP procedures.

### Adverse event reporting

All serious adverse events and specified non-serious adverse events occurring up until 28 days post-randomisation will be reported. Patients will carry a study card, which highlights the need to notify the recruiting GP/nurse regarding adverse events. Information about the adverse event will be transcribed into the patients’ medical records and reported to the SCTU as required.

Non-serious adverse events will only be recorded if due to any medical encounters related to the following:

- Chest symptoms: any events relating to cough, chest pain, or other symptoms of lower respiratory tract infection or their complications (including sepsis)
- Medication: any events relating to study medication

The general practitioners providing care for the patient are advised to record any event for which there is uncertainty as to whether it is study related or not, and to discuss with the chief investigator (CI).

### Sample size

The sample size for this study will be 160 patients overall or 40 patients per arm (4 arms in total) recruited from 20 sites. Eighty patients will receive the liquid formulation either active or placebo, and 80 will receive the tablet formulation either active or placebo in a 1:1 allocation ratio.

No formal sample size calculation was carried out; however, ignoring clustering, using a 95% confidence interval approach and an expected proportion of 50% of eligible patients randomised into the trial (to give the worst-case scenario), it can be shown that this sample size allows us to predict the recruitment rate to within 8% using nQuery Advisor v7.0.

Accounting for the clustering based on an intra-cluster correlation (ICC) of 0.05 and an expected proportion of 50% of eligible patients randomised into the trial, it can be shown that this sample size allows us to predict the recruitment rate (number of eligible patients randomised into the trial) to within 13%, given an average cluster size of 8 and 20 recruiting sites. Based on an ICC of 0.1, the sample size would allow us to predict the rate to within 14% [28].

### Statistical analysis plan

The analysis of this trial will be descriptive based on estimation rather than hypothesis testing, addressing the original trial objectives as set out in Table 1. All baseline measures and outcomes (e.g. recruitment, retention, and compliance) will be summarised for each allocated group using the appropriate descriptive statistics and presented with their associated confidence intervals. No formal comparison of groups will take place. A full statistical analysis plan will be developed prior to the final analysis of the trial. Consideration will be given to all of the experience and knowledge gained from running the HATRIC trial, including the trial team experience and the qualitative data as well as the quantitative data to make a conclusion about the feasibility of the main trial. The results of the trial will be reported in accordance with the CONSORT extension for pilot and feasibility trials [29].

Although no formal progression criteria were set at the start of this trial or written into the protocol, the feasibility of a definitive trial will be assessed against the objectives as set out in Table 1 alongside the data collected in the qualitative study:

- Is the recruitment rate per site per month adequate to make the required sample size for a definitive trial possible within a reasonable timeframe?
- Is the proportion of patients recruited from those eligible sufficient to allow the definitive trial results to be generalizable?
- Is the amount of data completion in the diaries sufficient to allow the definitive trial results to be generalizable?
- Do participants comply with either the liquid or the tablet intervention sufficiently to make a definitive trial worthwhile?
- Can we collect sufficient health economic data from participants so that a health economic analysis would be possible alongside the definitive trial?
- Is the rate of outcome measures in the control group compatible with conducting a definitive trial in the UK, with an achievable sample size, within a reasonable timeframe?

### Health economic analysis plan

We aim to develop and refine the methods for collecting resource use. The main resource usage will be the costs of the intervention and potential resource implications for the NHS including medication, primary care visits, outpatient attendance, A&E visits, and hospitalisation. Data on resource usage will be extracted mainly through case note review and from the patient diaries. Societal costs will be collected through the patient diary on out-of-pocket spending related to lower respiratory tract infection. These questions will be developed and tested during the feasibility study.

Quality of life will be measured by EQ-5D-5L. A utility score will be derived based on the UK tariff. The quality-adjusted life years (QALYS) will be calculated based on area under the curve approach. The QALYs estimated based on 2 point (baseline, 7 days) and 4 point (baseline, 2 days, 4 days, and 7 days) measurement will be compared.

The economic analysis of both costs and quality of life will be mainly descriptive and will be presented as means and standard deviations. Correlation of QALYs with main outcomes will be analysed to estimate sensitivity by different frequency of EQ-5D-5L measurements. The focus will be the direction of correlation and spread and confidence intervals.

Such information will allow us to establish for the definitive trial: the feasibility of estimating the costs of the intervention, the most relevant resource use data to collect, and the frequency that quality of life information should be collected.

### Ethical and regulatory aspects

The HATRIC study protocol has received the favourable opinion of the South Central–Berkshire B Research Ethics Committee. On advice from the UK Medicines and Healthcare products Regulatory Agency (MHRA), this feasibility study is not classed as a Clinical Trial of an Investigational Medicinal Product (CTIMP) because the outcome measures do not include efficacy of the product. A clinical trial authorisation (CTA) is not required for this feasibility trial.

Southampton Clinical Trials Unit (SCTU), an NIHR support funded CTU and UK Clinical Research Collaboration registered CTU, will coordinate the trial. A list of recruiting sites can be obtained from the SCTU. The University of Southampton is the sponsor for the trial (contact email: rgoinfo@soton.ac.uk). SCTU will communicate REC-approved protocol amendments to sites via email and updated trial documentation provided centrally via the trial website. Trial registries will be amended where relevant with explanations for these changes.

The HATRIC Trial Management Group (TMG) will include representatives from primary care, patient and public members, and SCTU staff involved in the day-to-day running of the trial. The TMG will be responsible for overseeing progress of the study, including both the clinical and practical aspects. The Chair of the TMG will be the chief investigator of the study.

The HATRIC trial will have an independent Trial Steering Committee (TSC) that will act as the oversight body on behalf of the sponsor and funder. The TSC will meet in person at least yearly and have at least one further teleconference meeting during the year. The majority of members of the TSC, including the Chair, will be independent of the trial. No Data Monitoring and Ethics Committee (DMEC) will be convened for HATRIC. This role will be assumed by the TSC.

This trial contributes to the University of Southampton antimicrobial resistant research strategy and is part of the Network for Anti-Microbial Resistance and Infection Prevention (NAMRIP) https://www.southampton.ac.uk/namrip/about/index.page.

### Patient and public involvement

The study has benefitted from patient and public involvement (PPI) from conception and through the design stage. PPI will continue throughout the conduct of the trial.

Patients and the public helped to design the trial through discussion of potential barriers to participation and the outcomes relevant to patients. The grant application was reviewed by two PPI representatives. They particularly highlighted the necessity for having plans in place to deal with the worry regarding the potential risks from untreated pneumonia with delayed antibiotics.

Furthermore, the trial has two PPI co-applicants who are full members of the TMG and who have provided additional advice on the trial design, the protocol, and all patient-facing study documentation. They will attend all TMG meetings.

We plan to utilise the skills of our PPI representatives to help interpret the qualitative data and to reflect on changes which may enhance recruitment and retention to the full trial if necessary. At the end of the study, it is important that the findings reach patient/public audiences and that the clinical audiences hear from the public voice; hence, we will include our PPI representatives in relevant presentations and/or articles to ensure maximum impact.

### Limitations

This trial is unable to include patients who have difficulty reading and understanding English. This decision was made due to funding and resourcing restrictions. Ideally, the trial would be able to include all patients who would, in practice, receive this care. This decision will impact on the generalisability of the trial results to the population of interest as a whole. We will summarise the patient mix at the end of study, to assess the potential impact of this criterion.

Furthermore, the HATRIC trial excluded women who were at risk of pregnancy. This decision was made, as there is no safety data currently for this patient group. The manufacturing authorisation in Germany for the Kaloba® preparation specifies that it must not be taken by women who are pregnant or breastfeeding. This should not affect the generalisability of the trial results, except as regards to pregnant and breastfeeding women.

## Discussion

If this trial demonstrates feasibility of recruitment and delivery, then we will seek funding for a fully powered placebo-controlled trial of *Pelargonium sidoides* root extract in primary care. Traditional herbal medicines have the potential to improve symptoms of common viral infections and hence reduce the symptom burden of these illnesses and to help reduce unnecessary antibiotic prescribing both nationally and internationally. Results will be disseminated to patients and clinical teams through peer-reviewed journal publications authored by the member of the TMG and presented at international conferences, with the help of the PPI representatives.

## Trial status

Recruitment opened on 14 March 2018 and is expected to be completed in December 2018. The current protocol is version 4, dated 13 August 2018.

## Data Availability

Pseudo-anonymised individual patient data (IPD) within the clinical trial dataset will be available for sharing via controlled access by authorised SCTU staff (as delegated to SCTU by the trial sponsor), and anonymised IPD within the clinical trial dataset will be available for sharing via open access after the trial is published. Data access can be requested via a SCTU data release application form, detailing the specific requirements and the proposed research, statistical analysis, publication plan, and evidence of research group qualifications. Data access requests are reviewed against specific eligibility criteria by the SCTU data custodian and key members of the trial team including a statistician and chief investigator or by an external independent review panel. Decisions about requests are made promptly and usually no more than 3 months after receipt of request. Details of all data requests and their outcomes, with clear rationale for any refusals are made swiftly back to the data requester.

## Abbreviations

A&E: Accident and emergency
CI: Chief investigator
COPD: Chronic obstructive pulmonary disease
CRN: Clinical Research Network
CRP: C-reactive protein
CTA: Clinical trial authorisation
CTIMP: Clinical Trial of an Investigational Medicinal Product
DMEC: Data Monitoring and Ethics Committee
eCRF: Electronic case report form
EQ-5D-5L: Five level, five dimension EuroQol questionnaire
GP: General practitioner
ICC: Intra-cluster correlation
IMP: Investigational medicinal product
IPD: Individual patient data
IRB: Institutional Review Board
ISF: Investigator Site File
LRTI: Lower respiratory tract infection
MHRA: Medicines and Healthcare products Regulatory Agency
NHS: National Health Service
NICE: National Institute for Health and Care Excellence
NIHR: National Institute for Health Research
PI: Principal investigator
PPI: Patient and public involvement
QALY: Quality-adjusted life year
REC: Research Ethics Committee
RTI: Respiratory tract infection
SCTU: Southampton Clinical Trials Unit
SOP: Standard operating procedure
SpO2: Peripheral capillary oxygen saturation
TMG: Trial Management Group
TSC: Trial Steering Committee
UK: United Kingdom
UoS: University of Southampton

## References

[1] Smith SM, Fahey T, Smucny J, Becker LA. Antibiotics for acute bronchitis. Cochrane Database Syst Rev. 2017;(6).

[2] Little P, Stuart B, Moore M, Coenen S, Butler CC, Godycki-Cwirko M (2013). Amoxicillin for acute lower-respiratory-tract infection in primary care when pneumonia is not suspected: a 12-country, randomised, placebo-controlled trial. Lancet Infect Dis.

[3] Moore M, Stuart B, Coenen S, Butler CC, Goossens H, Verheij TJ (2014). Amoxicillin for acute lower respiratory tract infection in primary care: subgroup analysis of potential high-risk groups. Br J Gen Pract.

[4] UK 5 Year Antimicrobial Resistance Strategy 2013 to 2018. Department of Health; 2013.

[5] HM Government. Tackling antimicrobial resistance 2019-2024: the UK’s five-year national action plan London: HM Government; 2019. Available from: https://www.gov.uk/government/publications/uk-5-year-action-plan-for-antimicrobial-resistance-2019-to-2024. 10.1016/j.jhin.2019.02.019 30826342

[6] Ashworth M, Charlton J, Ballard K, Latinovic R, Gulliford M (2005). Variations in antibiotic prescribing and consultation rates for acute respiratory infection in UK general practices 1995-2000. Br J Gen Pract..

[7] Gulliford MC, Dregan A, Moore MV, Ashworth M, Staa T, McCann G (2014). Continued high rates of antibiotic prescribing to adults with respiratory tract infection: survey of 568 UK general practices. BMJ Open..

[8] Pouwels KB, Dolk FCK, Smith DRM, Robotham JV, Smieszek T (2018). Actual versus ‘ideal’ antibiotic prescribing for common conditions in English primary care. J Antimicrob Chemother.

[9] Nice (2011). Prescribing of antibiotics for self-limiting respiratory tract infections in adults and children in primary care.

[10] National Institute for Clinical E (2014). NICE guidelines [CG191]: Pneumonia.

[11] Little P, Rumsby K, Kelly J, Watson L, Moore M, Warner G (2005). Information leaflet and antibiotic prescribing strategies for acute lower respiratory tract infection: a randomized controlled trial. JAMA..

[12] Little P, Moore M, Kelly J, Williamson I, Leydon G, McDermott L (2013). Ibuprofen, paracetamol, and steam for patients with respiratory tract infections in primary care: pragmatic randomised factorial trial. Br Med J.

[13] Llor C, Moragas A, Bayona C, Morros R, Pera H, Plana-Ripoll O (2013). Efficacy of anti-inflammatory or antibiotic treatment in patients with non-complicated acute bronchitis and discoloured sputum: randomised placebo controlled trial. Br Med J.

[14] Smith SM, Schroeder K, Fahey T (2012). Over-the-counter (OTC) medications for acute cough in children and adults in ambulatory settings. Cochrane Database Syst Rev..

[15] Wagner L, Cramer H, Klose P, Lauche R, Gass F, Dobos G (2015). Herbal medicine for cough: a systematic review and meta-analysis. Forsch Komplementmed..

[16] Timmer A, Gunther J, Motschall E, Rucker G, Antes G, Kern WV (2013). Pelargonium sidoides extract for treating acute respiratory tract infections. Cochrane Database Syst Rev..

[17] Excellence NIfHaC. Cough (acute): antimicrobial prescribing: NICE Guideline: draft for consultation; 2018.

[18] Kayser O, Kolodziej H (1997). Antibacterial activity of extracts and constituents of Pelargonium sidoides and Pelargonium reniforme. Planta Medica..

[19] Conrad A, Hansmann C, Engels I, Daschner FD, Frank U (2007). Extract of Pelargonium sidoides (EPs® 7630) improves phagocytosis, oxidative burst, and intracellular killing of human peripheral blood phagocytes in vitro. Phytomedicine..

[20] Kolodziej H, Kiderlen AF (2007). In vitro evaluation of antibacterial and immunomodulatory activities of Pelargonium reniforme, Pelargonium sidoides and the related herbal drug preparation EPs® 7630. Phytomedicine..

[21] Conrad A, Jung I, Tioua D, Lallemand C, Carrapatoso F, Engels I (2007). Extract of Pelargonium sidoides (EPs® 7630) inhibits the interactions of group A-streptococci and host epithelia in vitro. Phytomedicine..

[22] Rezaizadehnajafi L, Wink M (2014). Eps7630® from Pelargonium sidoides increases stress resistance in Caenorhabditis elegans probably via the daf-16/foxo pathway. Phytomedicine..

[23] Michaelis M, Doerr HW, Cinatl J (2011). Investigation of the influence of EPs® 7630, a herbal drug preparation from Pelargonium sidoides, on replication of a broad panel of respiratory viruses. Phytomedicine..

[24] Theisen LL, Muller CP (2012). EPs® 7630 (Umckaloabo®), an extract from Pelargonium sidoides roots, exerts anti-influenza virus activity in vitro and in vivo. Antivir Res.

[25] Watson L, Little P, Moore M, Warner G, Williamson I (2001). Validation study of a diary for use in acute lower respiratory tract infection. Fam Pract.

[26] Hay AD, Little P, Harnden A, Thompson M, Wang K, Kendrick D (2017). Effect of oral prednisolone on symptom duration and severity in nonasthmatic adults with acute lower respiratory tract infection: a randomized clinical trial. JAMA..

[27] Spurling GK, Del Mar CB, Dooley L, Foxlee R, Farley R. Delayed antibiotic prescriptions for respiratory infections. Cochrane Database of Syst Rev. 2017;(9):CD004417. 10.1002/14651858.CD004417.pub5 PMC6372405 28881007

[28] Eldridge SM, Costelloe CE, Kahan BC, Lancaster GA, Kerry SM (2016). How big should the pilot study for my cluster randomised trial be?. Stat Methods Med Res.

[29] Eldridge SM, Chan CL, Campbell MJ, Bond CM, Hopewell S, Thabane L (2016). CONSORT 2010 statement: extension to randomised pilot and feasibility trials. Pilot Feasibility Stud.

[30] Chan Carusone SB, Walter SD, Brazil K, Loeb MB (2007). Pneumonia and lower respiratory infections in nursing home residents: predictors of hospitalization and mortality. J Am Geriatr Soc.

[31] Mehr DR, Binder EF, Kruse RL, Zweig SC, Madsen R, Popejoy L (2001). Predicting mortality in nursing home residents with lower respiratory tract infection: the Missouri LRI study. JAMA..

[32] Chan A-W, Tetzlaff JM, Altman DG, Laupacis A, Gøtzsche PC, Krleža-Jerić K (2013). SPIRIT 2013 statement: defining standard protocol items for clinical trials. Ann Intern Med.

